# Supplementary material for: Rapid and quantitative phage susceptibility test by ramanome
Source: mLife. 2026 May 21;5(3):369–82. doi: 10.1002/mlf2.70089 (PMC13327610; doi:10.1002/mlf2.70089)
Supplement: Supplementary file 1 — Supplementary Materials 20260512. [file MLF2-5-369-s001.pdf]

Supporting information to: Rapid and Quantitative Phage Susceptibility Test by Ramanome

Xiao Han[1,2,#], Xiaofu Wan[3,#], Yang Zhou[3,4], Xiaoting Fu[2], Xiaoshan Zheng[2,5], Bo Gao[2,6], Shi Huang[7], Anle Ge[2,5], Jiadong Huang[8,*], Hongzhou Lu[3,*], Jian Xu[2,5,*]

[1]School of Chemistry and Chemical Engineering, University of Jinan, Jinan, China

[2]Single-Cell Center, Shandong Energy Institute, Qingdao New Energy Shandong Laboratory, Qingdao Institute of Bioenergy and Bioprocess Technology, Chinese Academy of Sciences, Qingdao, China

[3]National Clinical Research Center for Infectious Diseases, Shenzhen Clinical Medical Research Center for Tuberculosis, Shenzhen Third People's Hospital, Southern University of Science and Technology, Shenzhen, China

[4]Molecular Biology Research Center & Center for Medical Genetics, School of Life Sciences, Central South University, Changsha, China

[5]University of Chinese Academy of Sciences, Beijing, China

[6]Single-Cell Biotechnology Ltd., Qingdao, China

[7]Faculty of Dentistry, University of Hong Kong, Hong Kong, China

[8]School of Life Sciences, University of Jinan, Jinan, China

[#]These authors contributed equally to this work.

*Corresponding to: xujian@qibebt.ac.cn, luhongzhou@fudan.edu.cn, and chm_huangjd@ujn.edu.cn

This PDF file includes:

Supplementary Tables (S1, S2)

Supplementary Figures (S1 to S3)

References (1 to 10)

Other Supplementary Materials for this manuscript include the following:

Supplementary Data S1

Supplemental Tables

Supplemental Table 1. Biological assignments of SCRS.

Raman shift （cm^{-1})	Biomolecule assignment	Molecular vibration	Reference
666	Protein	Cysteine C-S stretching vibration	(1)
669	Nucleic acids	Guanine ring breathing modes	(2)
719	Phospholipids	C-N stretching vibration	(3)
723	Nucleic acids	Adenine ring breathing mode	(4)
780	Nucleic acids	Ring breathing modes of cytosine, thymine, and uracil	(5)
804	Nucleic acids	Stretching vibrations of O-P-O	(6)
843	Glucose	CH rocking	(7)
871	Protein	Single bond stretching vibrations for the proline and valine	(8)
993	Protein	Phenylalanine ring breathing mode	(9)
1023	Glycogen	Carbohydrates Deformation vibration of C-O-H	(7)
1094	Nucleic acids	Symmetric phosphate stretching vibration	(5)
1113	Protein	Benzoid ring deformation	(8)
1242	Nucleic acids	C-N in-plane stretching	(3)
1285	Lipids	C=C stretching mode	(8)
1330	Nucleic acids	CH_3CH_2 wagging	(3)
1369	Phospholipids	CH_3 stretching mode	(4)
1408	Lipids	CH_2 stretching mode	(9)
1444	Lipids, protein	CH_2 scissoring mode	(6, 8)
1471	Lipids	CH_2 bending mode	(6)
1476	Nucleic acids	Nucleotide acid purine bases	(3)
1525	Carotenoid	C=C stretching mode	(10)
1574	Nucleic acids	Ring breathing modes of bases	(5)
1595	Protein	Phenylalanine C=C skeletal vibration	(6)
1676	Nucleic acids	Ring breathing modes of cytosine, thymine, and uracil	(8)

Supplemental Table 2. Comparison of S/R derived from Plaque assay and RPST.

Species	Strain	Phage	Susceptible (S) /Resistant (R)		MEM_RPST*
			Plaque assay	RPST	
	ATCC11303	T1	S	S	0.01
		T4	S	S	0.01
	ATCC25922	T4	S	S	10
		T1	R	R	> 10
		Ecp2	S	S	< 10
		Ecp5	S	S	< 10
		Ecp10	R	R	> 10
E. coli		Ecp11	S	S	< 10
		Ecp19	S	S	< 10
	DH5*α*	Ecp44	S	S	< 10
		Ecp91	S	S	< 10
		Ecp101	R	R	> 10
		Ecp9	S	S	0.1
		Ecp32	S	S	0.01
		Ecp54	S	S	0.1
		Ecp12	R	R	> 10
	Sal8		S	S	1
S. enterica	Sal28	Salp6	S	S	< 10
	Sal2		R	R	> 10
	Pae1		S	S	0.01
P. aeruginosa	Pae307	Pap12	S	S	1
	Pae5		R	R	> 10
	Kpn25		S	S	0.1
K. pneumoniae	Kpn30	Kpnp4	S	R [ϕ]	> 10
	Kpn6		R	R	> 10

* MEM_RPST: the minimum effective MOI of phages based on RPST.

ϕ Gray-shaded cells indicate cases where RPST and plaque assay results were discordant.

Supplemental Figures

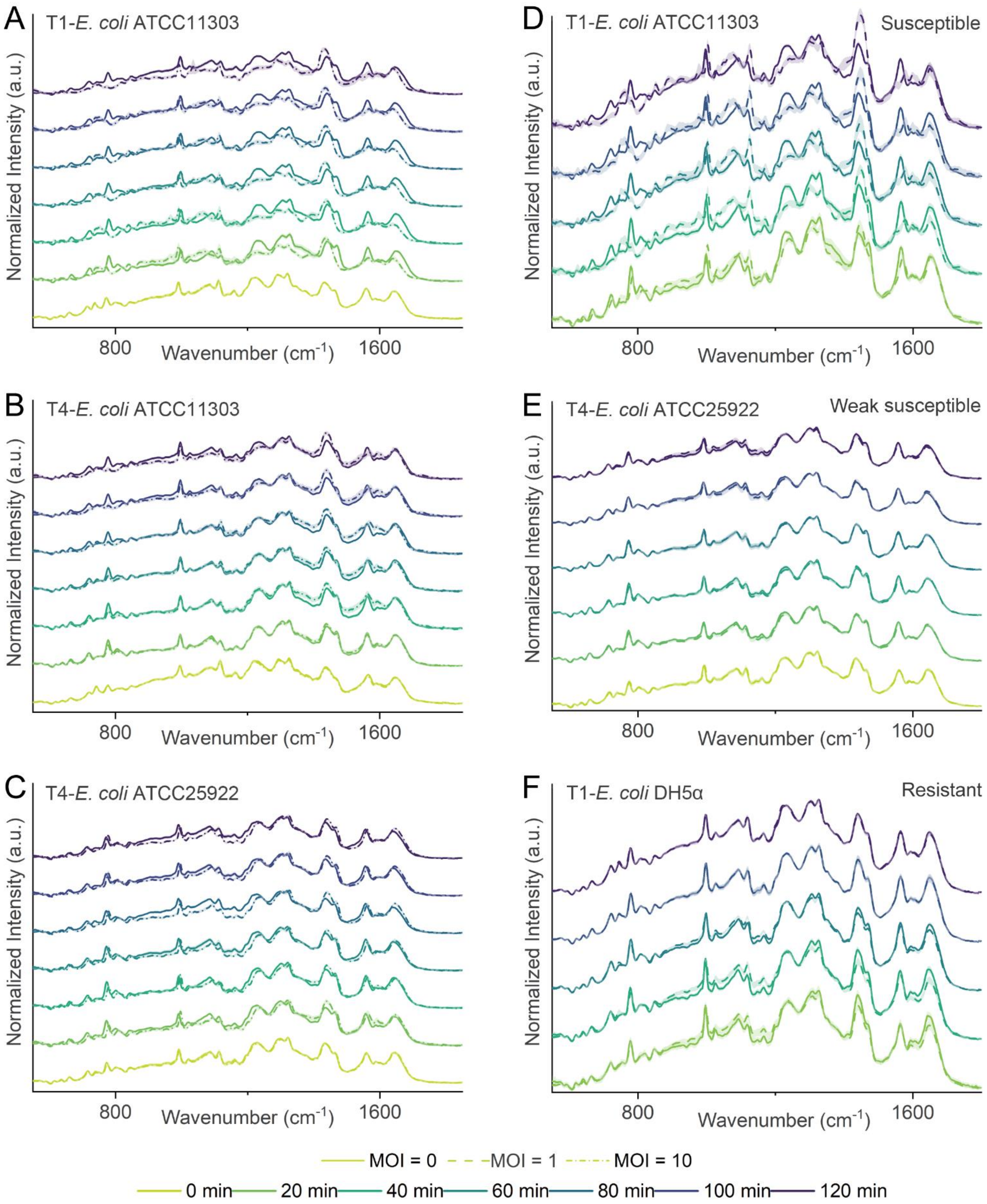

Figure S1. Population-level Ramanome dynamics during phage–host co-incubation. Ramanomes were collected from bacteria co-incubated with different phages to visualize overall spectral changes

over time. (**A-C**) Spectral comparisons between infected (MOI = 10) and uninfected (MOI = 0) groups in three susceptible systems: (**A**) T1-*E. coli* ATCC11303, (**B**) T4-*E. coli* ATCC11303, and (**C**) T4-*E. coli* ATCC25922, measured at seven time points (0–120 min, 20-min intervals). (**D-F**) Spectral comparisons between experimental (MOI = 1) and control (MOI = 0) groups in three systems: (**D**) T1-*E. coli* ATCC11303, (**E**) T4-*E. coli* ATCC25922, and (**F**) T1-*E. coli* DH5α, measured at five time points (20–100 min, 20-min intervals).

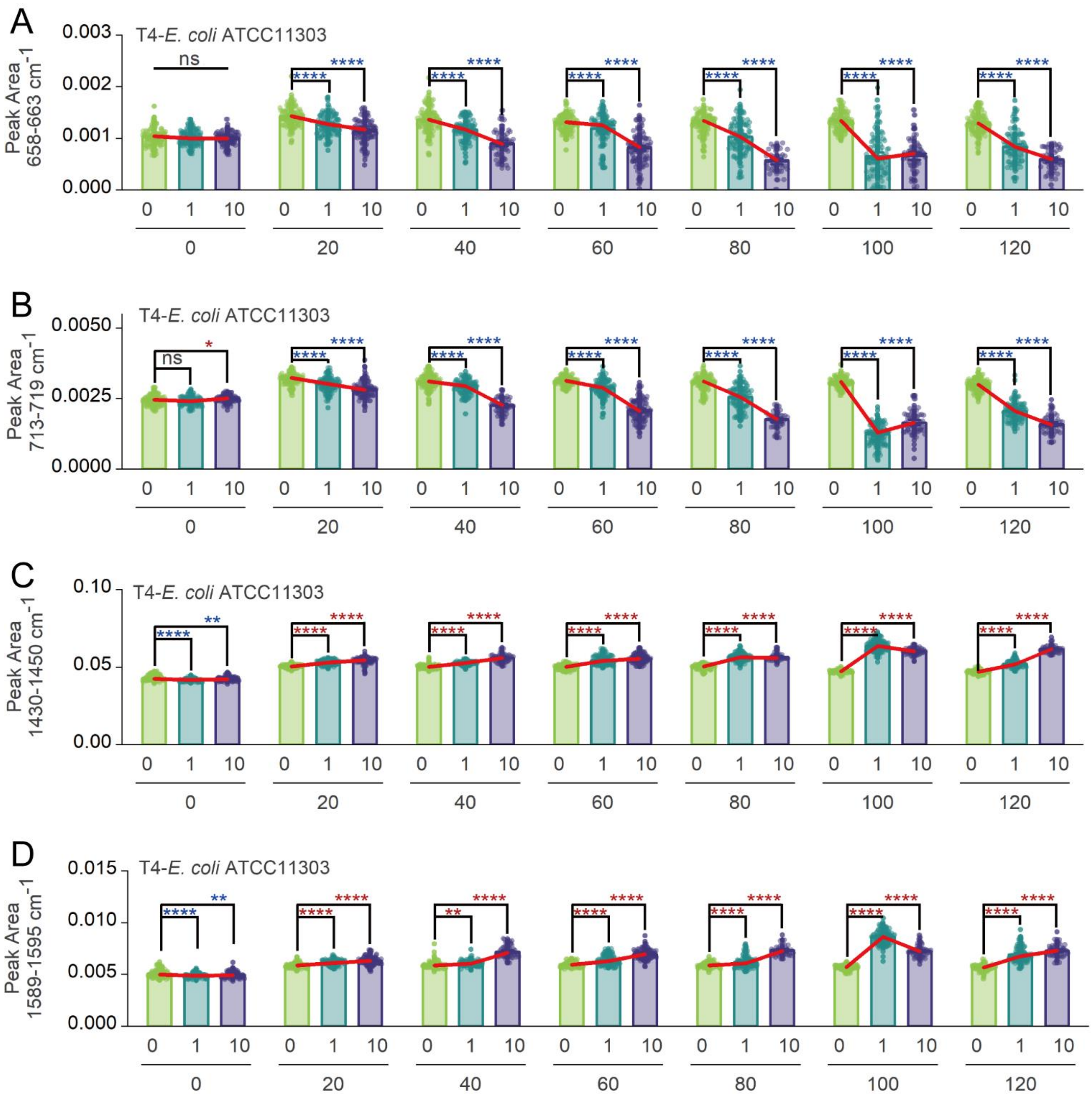

Figure S2. Consistent time-dependent shifts of four Raman biomarkers in a highly susceptible system. *E. coli* ATCC11303 were co-incubated with T4 phage at MOI = 0, 1, or 10, and ramanomes were collected at 0, 20, 40, 60, 80, 100, and 120 min. In the infected groups, the relative intensities of (**A**) 658–663 cm^{-1} and (**B**) 713–719 cm^{-1} decrease starting from 20 min post-infection, with the decline becoming more pronounced over time (observed at both MOI = 1 and MOI = 10). In contrast, the relative intensities of (**C**) 1430–1450 cm^{-1} and (**D**) 1589–1595 cm^{-1} begin to increase from 20 min post-infection,

with the increase becoming more pronounced over time (also observed at MOI of 1 and 10). Statistical analysis was performed using two-sided Student's *t*-test. ns, not significant; $*p < 0.05$, $**p < 0.01$, $***p < 0.001$, $****p < 0.0001$. Red asterisks indicate that the statistical value is higher in the experimental group than in the control group, while blue asterisks indicate lower.

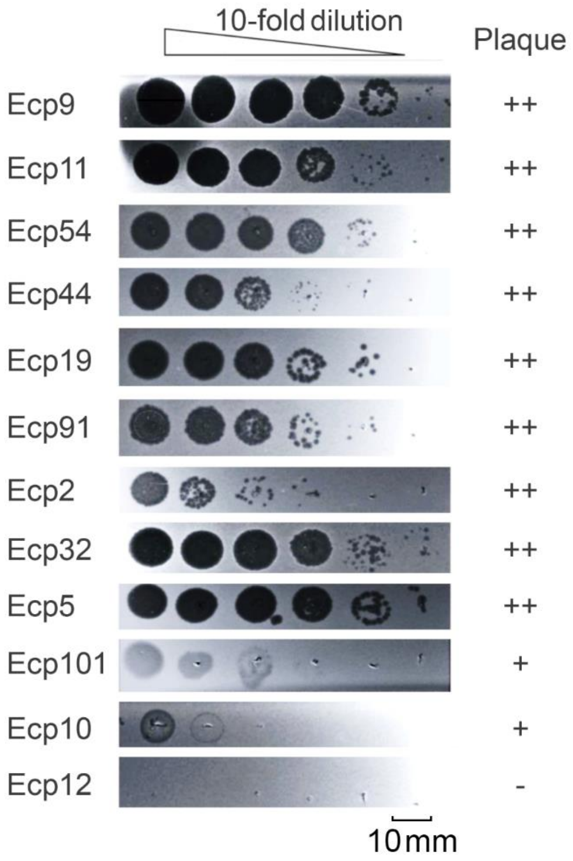

Figure S3. Plaque assay outcomes of twelve lytic phages on *E. coli* DH5*α*. Nine phages produce clear and well-defined plaques on *E. coli* DH5*α* via agar plates, whereas the other three yield turbid or indistinct plaques or no visible plaques at all.

Reference

1. Teng L, Wang X, Wang X, Gou H, Ren L, Wang T, et al. Label-free, rapid and quantitative phenotyping of stress response in *E. coli* via ramanome. Sci Rep. 2016;6:34359.
2. Indra M, Victoria T, Sarah P E, André S, Lara I T, Katrin W, et al. Label-free Raman microspectroscopy for identifying prokaryotic virocells. mSystems. 2022;7:e0150521.
3. Guo G, Guo C, Qie X, He D, Meng S, Su L, et al. Correlation analysis between Raman spectral signature and transcriptomic features of carbapenem-resistant *Klebsiella pneumoniae*. Spectrochim Acta A Mol Biomol Spectrosc. 2024;308:123699.
4. Zheng X, Li R, Wang T, Li X, Han X, Dai Y, et al. Unraveling antibacterial mechanisms of surfactants against *Staphylococcus aureus* via single-cell Raman spectroscopy. Anal Chem. 2025;97:9202-11.
5. Yang K, Xu F, Zhu L, Li H, Sun Q, Yan A, et al. An Isotope-labeled single-cell Raman spectroscopy approach for tracking the physiological evolution trajectory of bacteria toward antibiotic resistance. Angew Chem Int Ed Engl. 2023;62:e202217412.
6. Garg A, Nam W, Wang W, Vikesland P, Zhou W. In situ spatiotemporal SERS measurements and multivariate analysis of virally infected bacterial biofilms using nanolaminated plasmonic crystals. ACS Sens. 2023;8:1132-42.
7. Mohacek-Grosev V, Brljafa S, Skrabic M, Maric I, Blazek Bregovic V, Amendola V, et al. Glucosamine to gold nanoparticles binding studied using Raman spectroscopy. Spectrochim Acta A Mol Biomol Spectrosc. 2022;264:120326.
8. Movasaghi Z, Rehman S, Rehman IU. Raman spectroscopy of biological tissues. Appl Spectrosc Rev. 2007;42:493-541.
9. Ito H, Uragami N, Miyazaki T, Shimamura Y, Ikeda H, Nishikawa Y, et al. Determination of esophageal squamous cell carcinoma and gastric adenocarcinoma on raw tissue using Raman spectroscopy. World J Gastroenterol. 2023;29:3145-56.
10. Dhankhar D, Nagpal A, Li R, Chen J, Cesario TC, Rentzepis PM. Resonance Raman spectra for the in situ identification of bacteria strains and their inactivation mechanism. Appl Spectrosc. 2021;75:1146-54.

Data S1. (separate file)

Ramanome-based Composite Infection Index (CII) Dataset.

This spreadsheet contains the analyzed ramanomic data for all bacterium-phage co-incubation systems investigated in this study. It reports the peak areas of the four characteristic Raman biomarkers used for the CII model, alongside the computed CII scores, predicted labels, confidence values, and predicted status for each spectrum based on the model output. The file also provides the final sample-level infection call, which is determined by aggregating the CII results from all ramanomes per sample.

The dataset is organized into multiple sheets: The first sheet, titled "Result_sum", provides a summary of the results at the sample level. Subsequent sheets are dedicated to the single-spectrum level data for each unique co-incubation system, named according to the convention: BacterialStrain_Phage (e.g., ATCC11303_T4).
